# Supplementary material for: Assessing the effectiveness of empirical calibration under different bias scenarios
Source: BMC Med Res Methodol. 2022 Jul 27;22:208. doi: 10.1186/s12874-022-01687-6 (PMC9327283; doi:10.1186/s12874-022-01687-6)

Supplementary Material

1 DATA GENERATION DIRECTED ACYCLIC GRAPH 2

2 MODELLING THE SYSTEMATIC ERROR IN EMPIRICAL CALIBRATION 3

3 RUNNING SIMULATION CODE TO REPLICATE RESULTS 5

4 COVERAGE PLOTS OF CALIBRATION OF CONTROLS 6

4.1 UNMEASURED CONFOUNDER SCENARIO 7

4.1.1 Calibration of Negative Controls (Five) 7

4.1.2 Calibration of Positive Controls (Five) 8

4.1.3 Calibration of Negative Controls (30) 9

4.1.4 Calibration of Positive Controls (30) 10

4.2 QUADRATIC TERM SCENARIO 11

4.2.1 Calibration of Negative Controls (Five) 11

4.2.2 Calibration of Positive Controls (Five) 12

4.2.3 Calibration of Positive Controls (30) 14

4.3 INTERACTION BETWEEN TWO CONFOUNDERS 15

4.3.1 Calibration of Negative Controls (5) 15

4.3.2 Calibration of Positive Controls (5) 16

4.3.3 Calibration of Negative Controls (30) 17

4.3.4 Calibration of Positive Controls (30) 18

4.4 LACK OF POSITIVITY SCENARIO 19

4.4.1 Calibration of Negative Controls (5) 19

4.4.2 Calibration of Positive Controls (5) 20

4.4.3 Calibration of Negative Controls (30) 21

4.4.4 Calibration of Positive Controls (30) 22

4.5 MEASUREMENT ERROR IN CONFOUNDER SCENARIO 23

4.5.1 Calibration of Negative Controls (5) 23

4.5.2 Calibration of Positive Controls (5) 24

4.5.3 Calibration of Negative Controls (30) 25

4.5.4 Calibration of Positive Controls (30) 26

1 Data generation directed acyclic graph

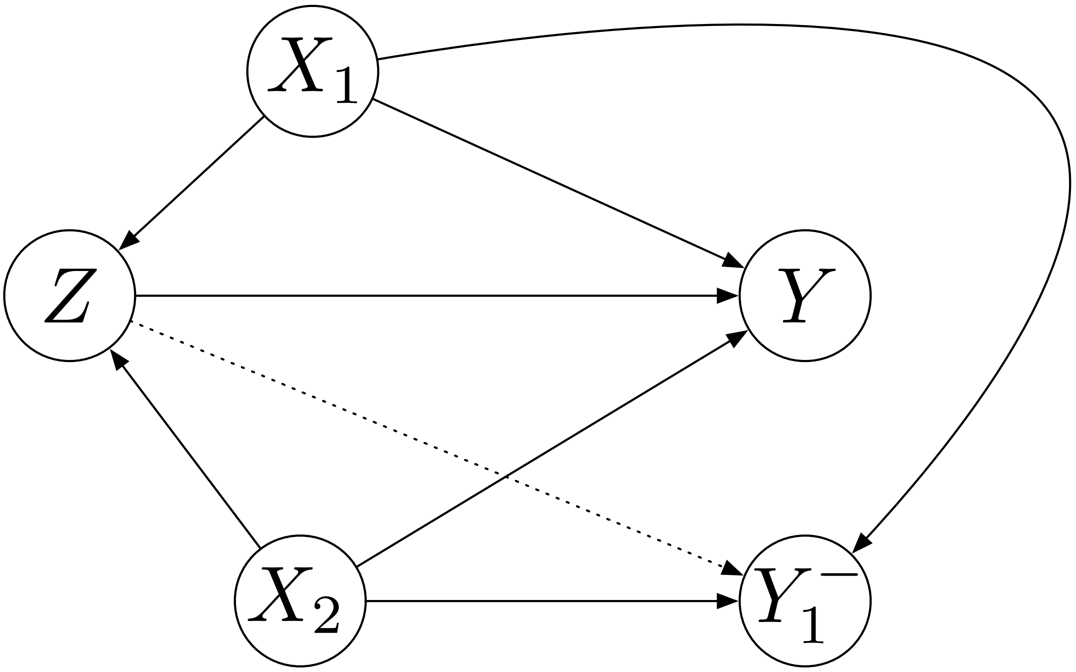

Figure 1. Direct acyclic graph of simulation setup with outcome of interest Y, one negative control outcome Y_1^- and two measured confounders X_1 and X_2. The dashed line between the treatment Z and the negative control indicates a potential association, for an ideal negative control.

2 Modelling the systematic error in empirical calibration

Suppose there are a total of S negative control outcomes, each with V corresponding number of positive controls. Let denote $\theta_i, i \in \{1,2,\ldots,S \times V\}$ as the treatment effect of each individual control with $\theta_i = 0$ for negative controls and $\theta_i > 0$ for positive controls. An estimate of the treatment effect can be modelled as

$$\widehat{\theta_i} = \theta_i + \epsilon_i \quad , \quad \epsilon_i \sim \mathcal{N}(0, \tau^2)$$

such that $\widehat{\theta_i} - \theta_i$ is due to the random error term ϵ_i.

Empirical calibration assumes that there is a *systematic bias* component to the error term, (here denoted by ψ_i such that

$$\epsilon_i \sim \mathcal{N}(\psi_i, \tau^2).$$

The bias ψ_i is assumed to come from another Gaussian distribution

$$\psi_i \sim \mathcal{N}\{\mu(\theta_i), \sigma^2(\theta_i)\}$$

with the mean and variance estimated as parametric functions of θ_i

$$\mu(\theta_i) = \alpha + \gamma\theta_i \quad (1)$$

$$\log\{\sigma(\theta_i)\} = \phi + \nu\theta_i \quad (2)$$

The parameters in Equation 1 and Equation 2 α,γ,ϕ,ν are estimated by maximising the likelihood of observing the joint probability distribution of the systematic bias ψ_i and estimates from all controls $\widehat{\theta_i}$

$$\Pr(\widehat{\theta_i}, \psi_i) = \Pr(\widehat{\theta_i} \mid \psi_i)\Pr(\psi_i). \quad (3)$$

Since we do not observe ψ_i, we integrate them out from Equation 3 to obtain the marginal probability distribution of our estimates $\widehat{\theta_i}$ as a function of the known θ_i

$$\Pr(\widehat{\theta_i}) = \int \Pr(\widehat{\theta_i}, \psi_i)\, d\psi_i.$$

The likelihood of this marginal distribution is then given by

$$\mathcal{L}(\alpha,\gamma,\phi,\nu) \sim \propto \prod_{i=1}^{S\times V} \int \Pr(\theta_i \mid \psi_i, \theta_i, \hat{\tau}_i)\Pr(\psi_i \mid \alpha,\gamma,\phi,\nu,\theta_i)\, d\psi_i$$

A new estimate is then assumed to come from an empirical Gaussian distribution which incorporates the estimate error. See [1-2] for more detail.

References

1. Schuemie MJ, Hripcsak G, Ryan PB, Madigan D, Suchard MA. Empirical confidence interval calibration for population-level effect estimation studies in observational healthcare data. Proceedings of the National Academy of Sciences of the United States of America. 2018;115(11):2571-7.
2. Schuemie MJ, Ryan PB, Pratt N, Chen R, You SC, Krumholz HM, et al. Large-scale evidence generation and evaluation across a network of databases (LEGEND): assessing validity using hypertension as a case study. Journal of the American Medical Informatics Association. 2020;27(8):1268-77.

3 Running simulation code to replicate results

The source code used to run our simulations and instructions on replicating the results and figures from this paper are in GitHub: https://github.com/clinical-ai/assess-empcalib

The flowchart in Figure 2 illustrates the primary steps followed in our simulation to generate the data, estimate treatment effects on the outcome of interest, generating the positive controls, and performing empirical calibration. This is repeated for a number of iterations, with the results from these iterations used to generate the funnel plots presented in the Results section.

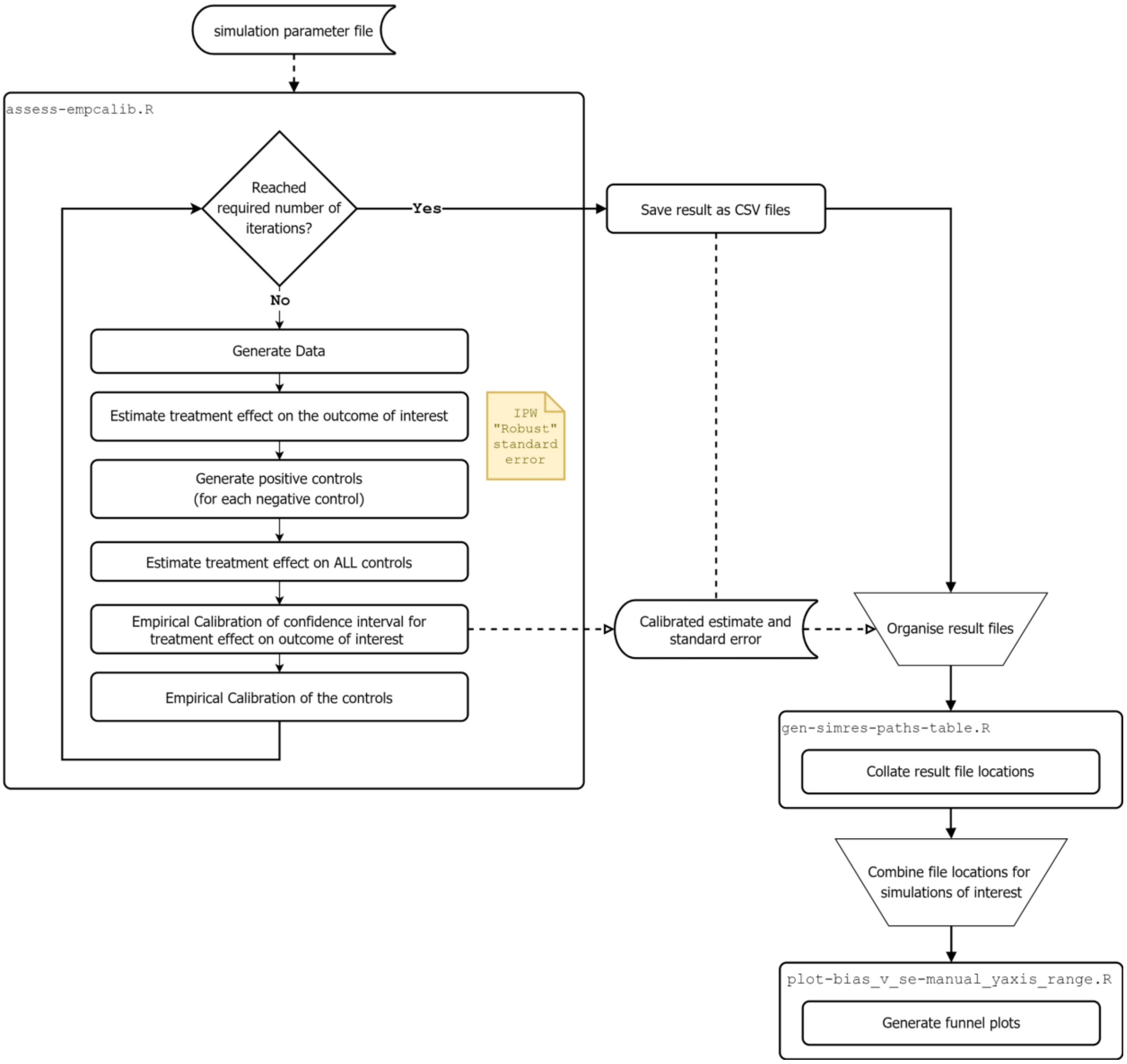

Figure 2. Flowchart detailing the primary execution steps of our simulation.

4 Coverage plots of calibration of controls

For each bias simulation scenario, coverage plots of uncalibrated and calibrated estimates and corresponding standard errors for positive and negative controls are shown. The estimates are stratified by true effect size.

4.1 Unmeasured Confounder Scenario

4.1.1 Calibration of Negative Controls (Five)

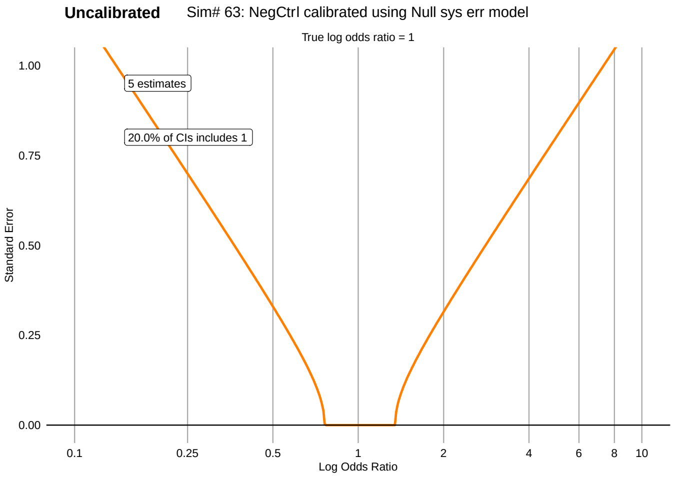

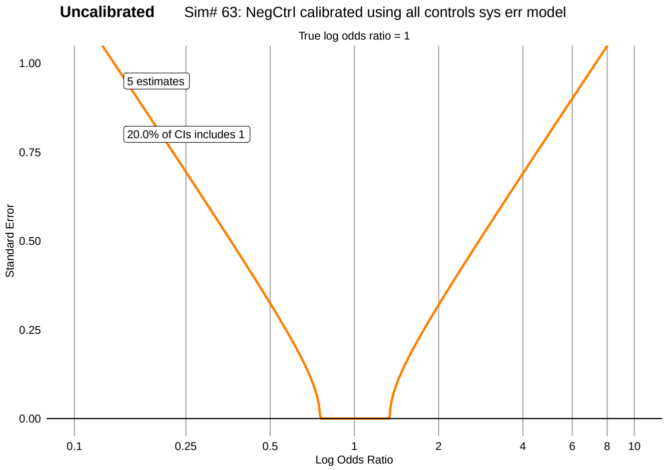

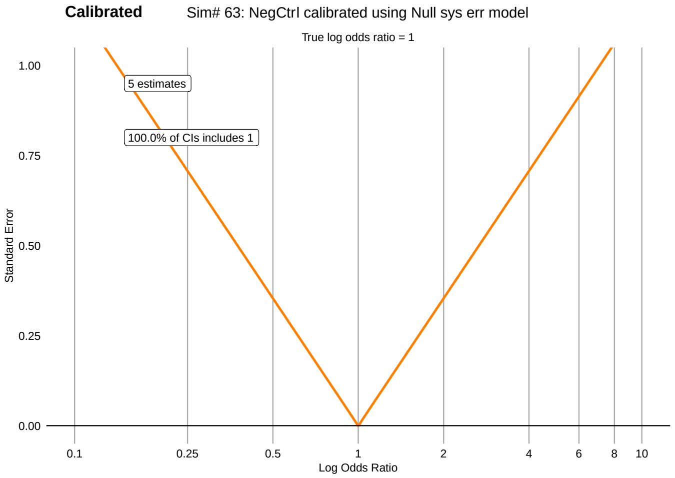

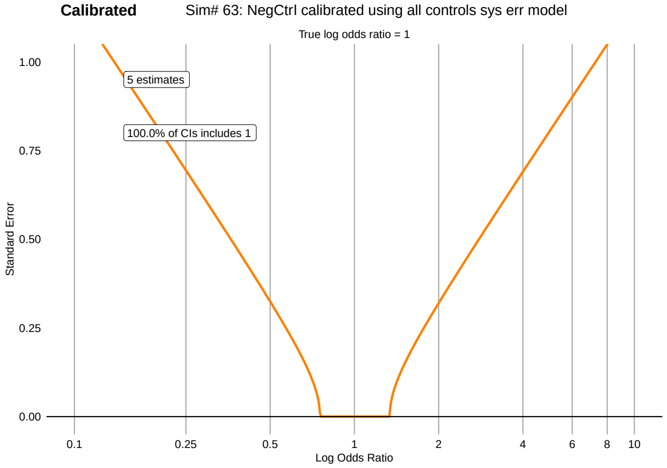

4.1.2 Calibration of Positive Controls (Five)

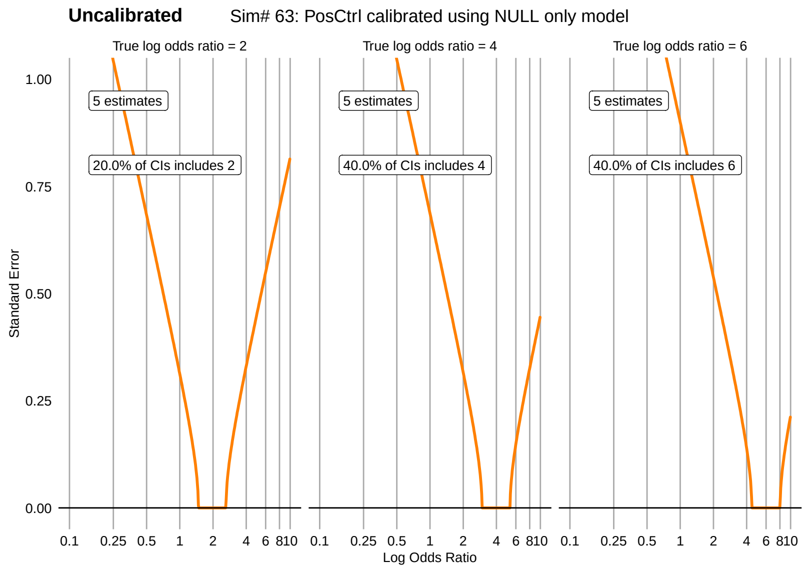

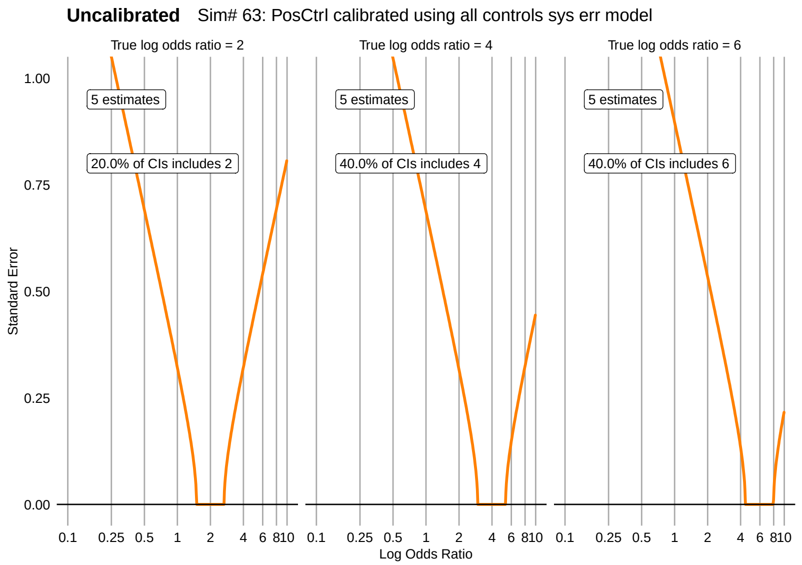

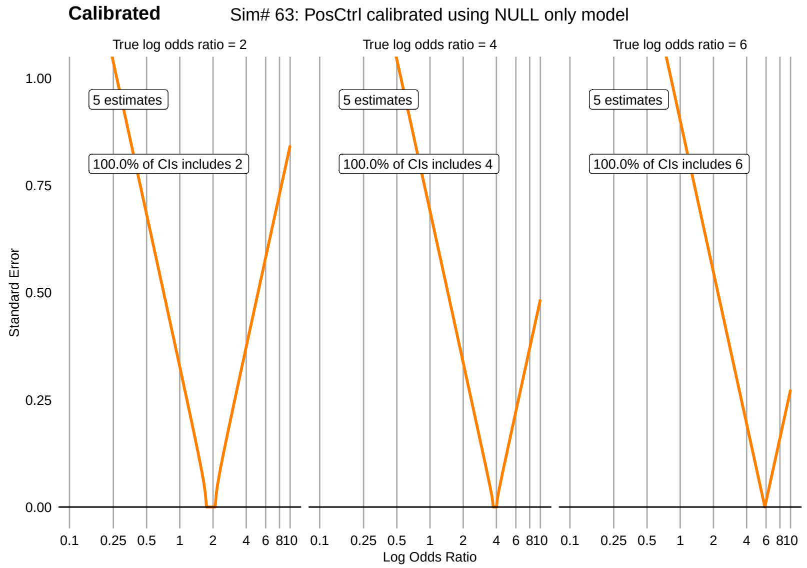

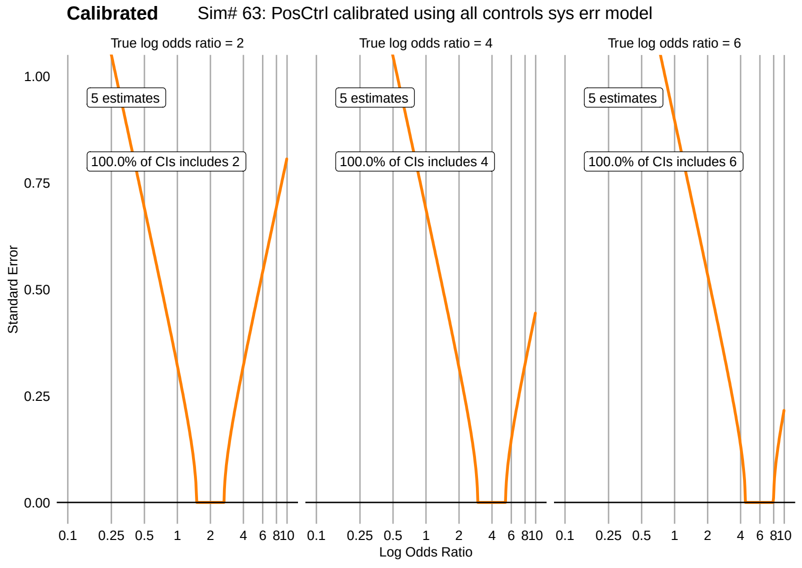

4.1.3 Calibration of Negative Controls (30)

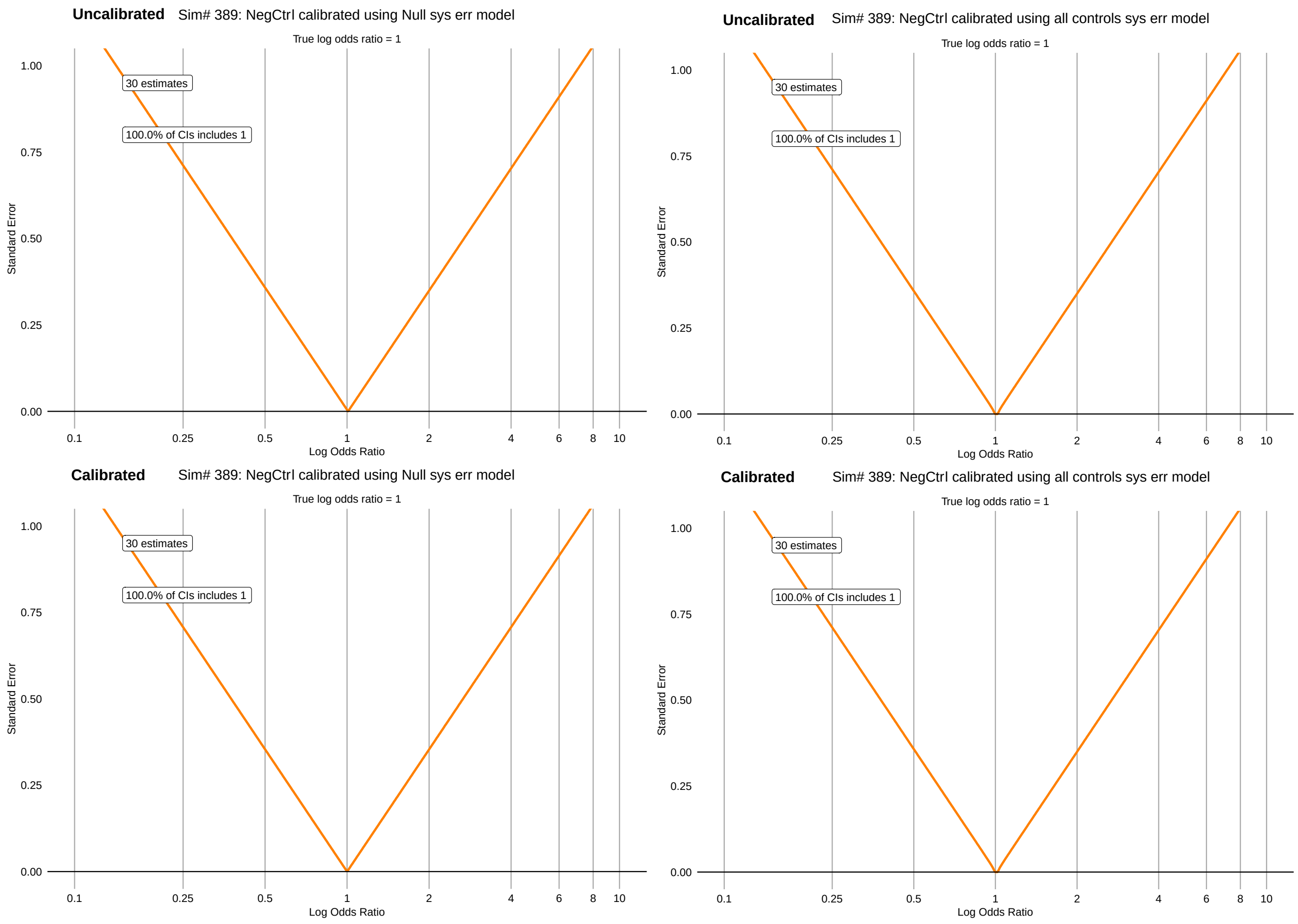

4.1.4 Calibration of Positive Controls (30)

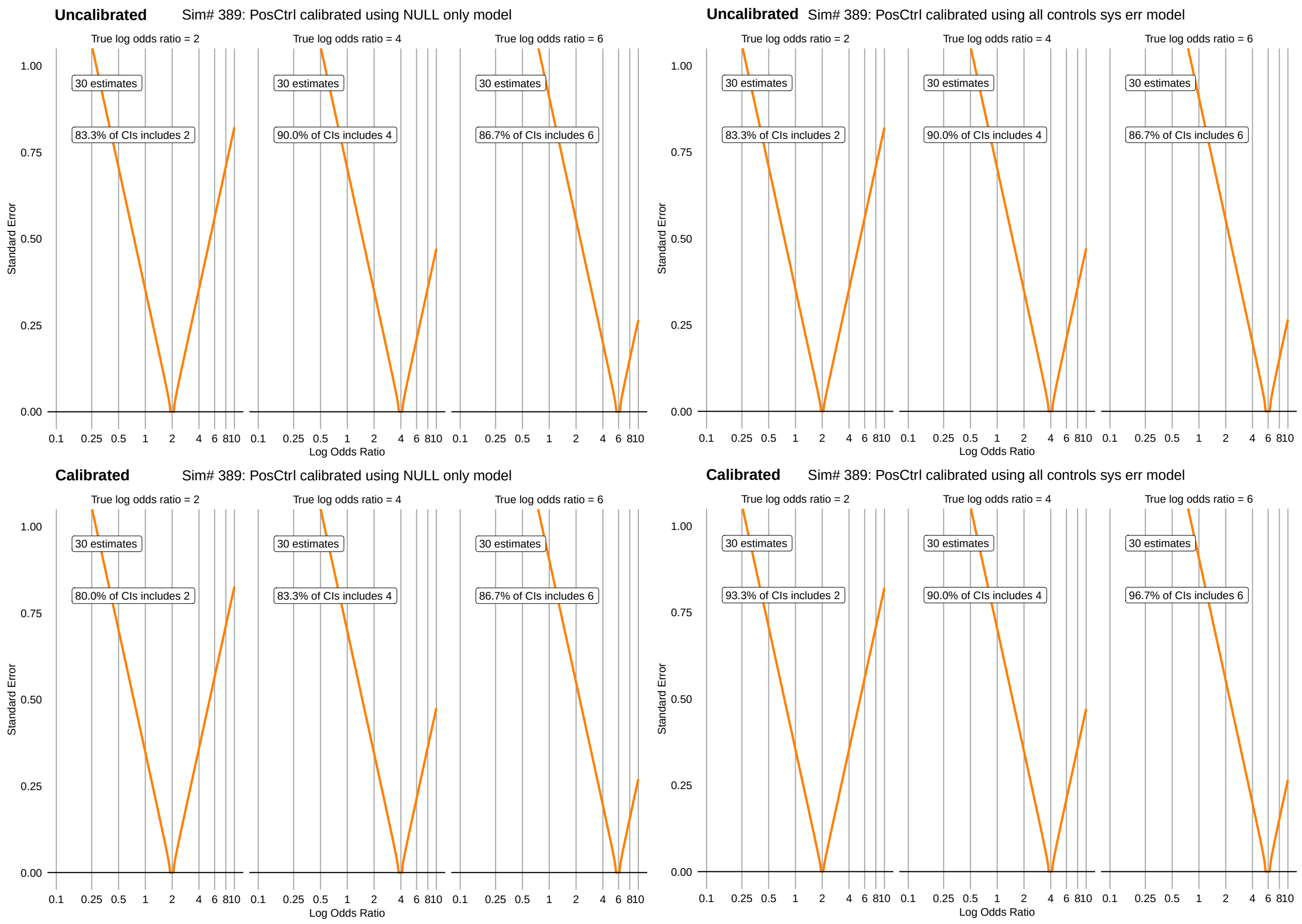

4.2 Quadratic Term Scenario

4.2.1 Calibration of Negative Controls (Five)

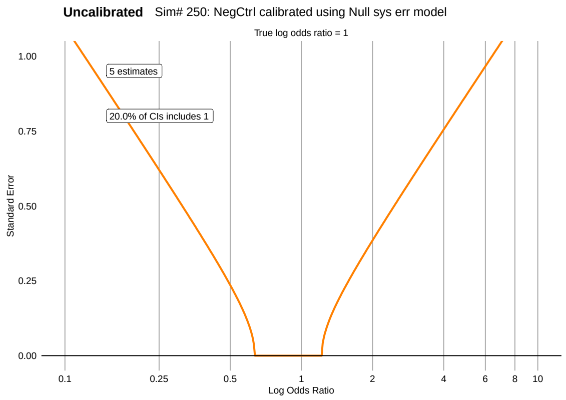

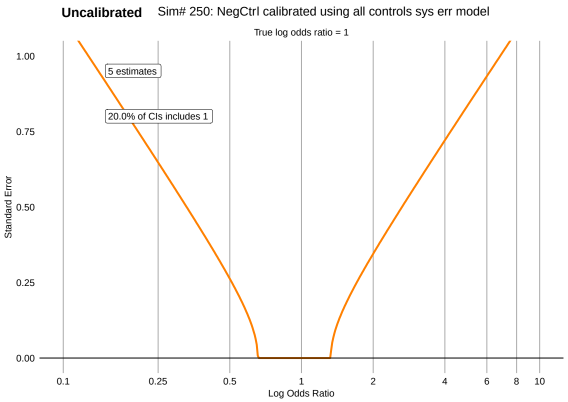

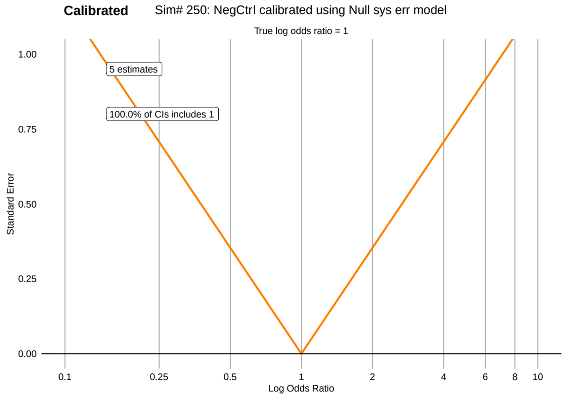

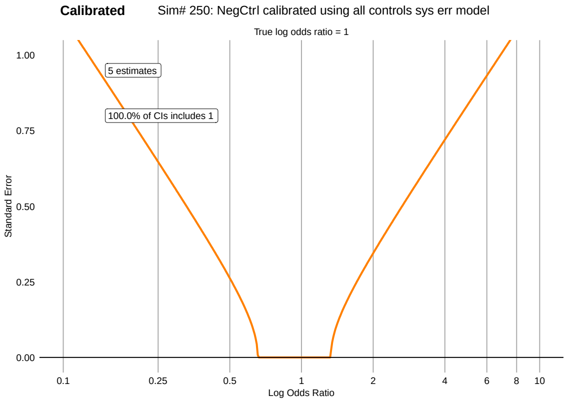

4.2.2 Calibration of Positive Controls (Five)

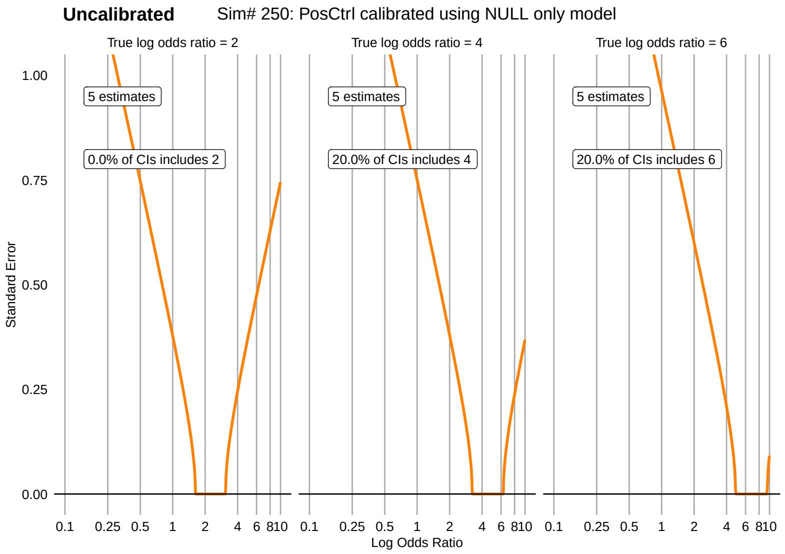

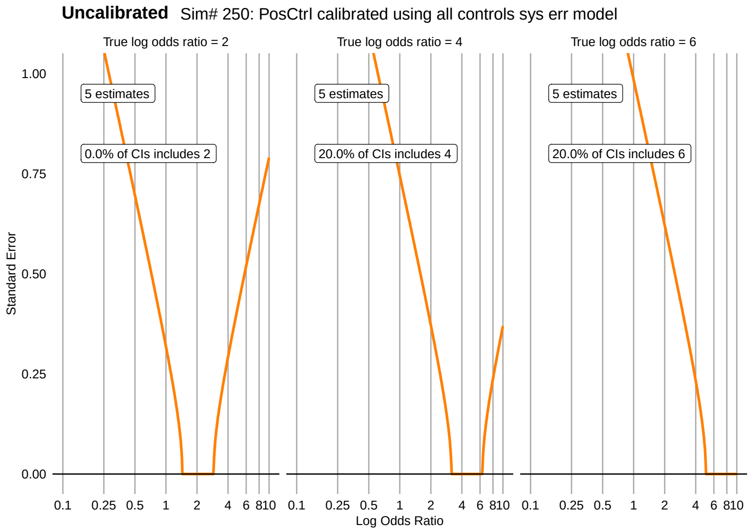

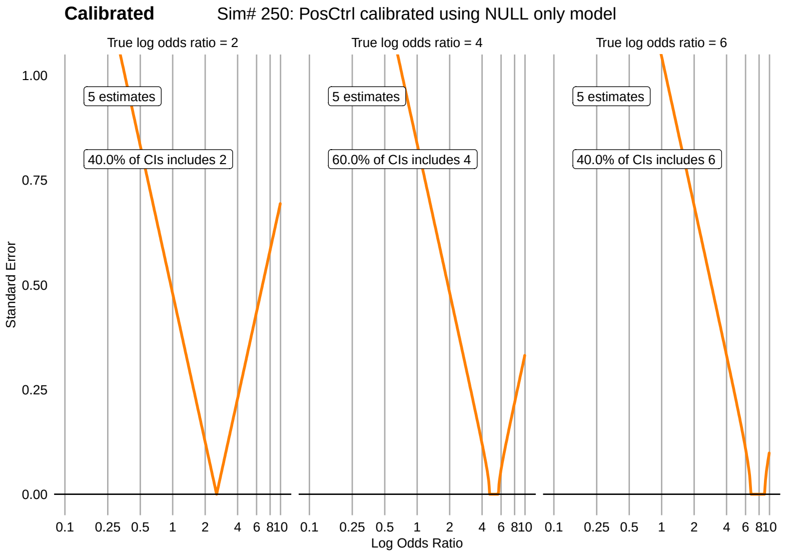

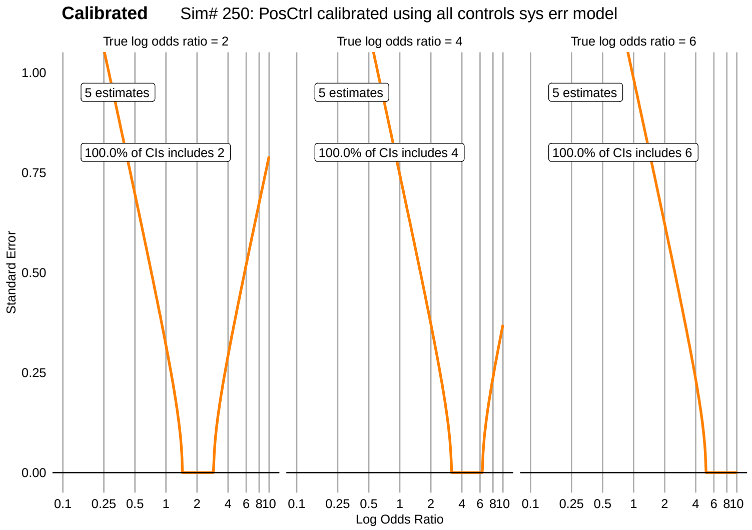

Calibration of Negative Controls (30)

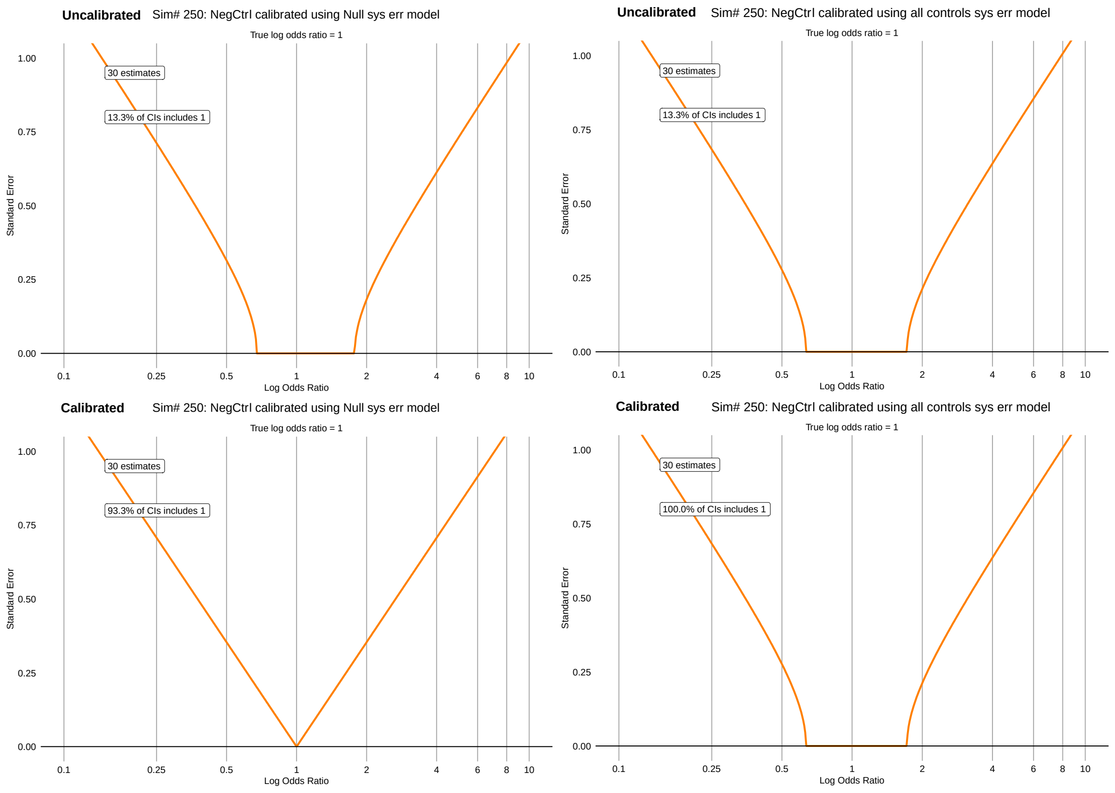

4.2.3 Calibration of Positive Controls (30)

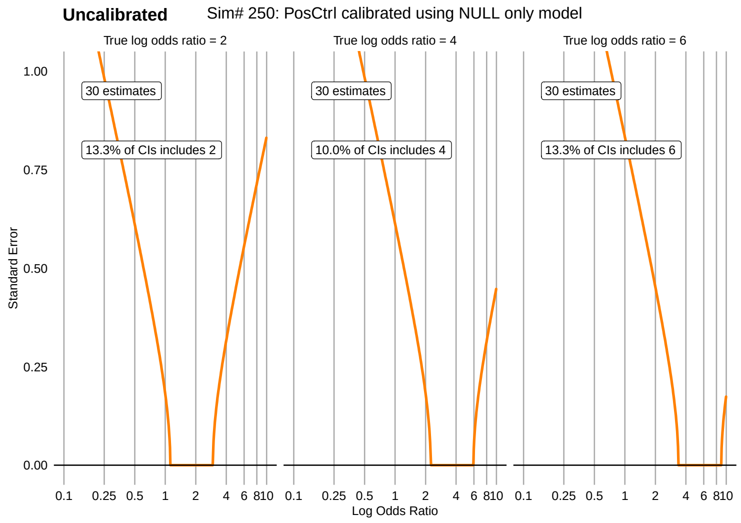

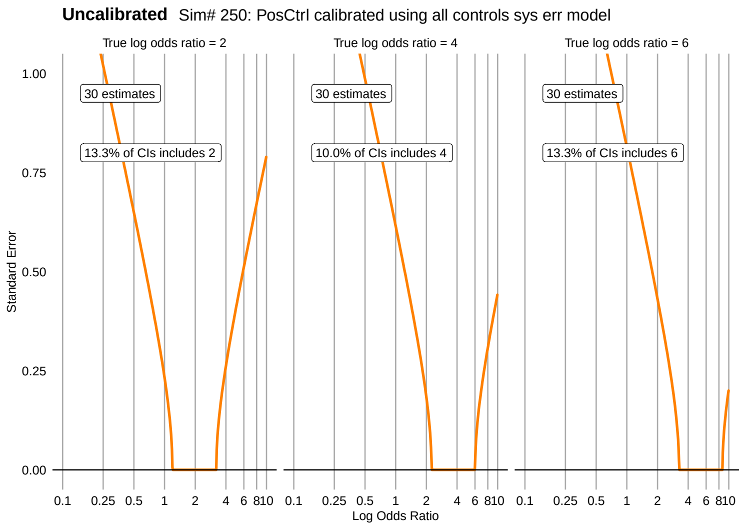

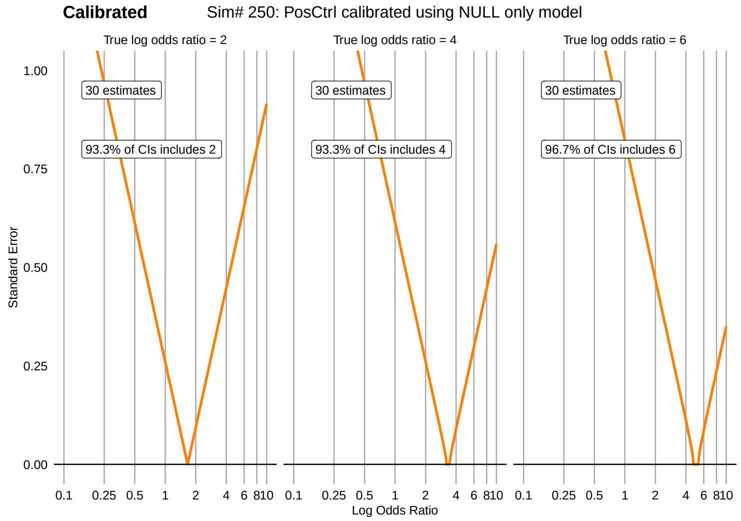

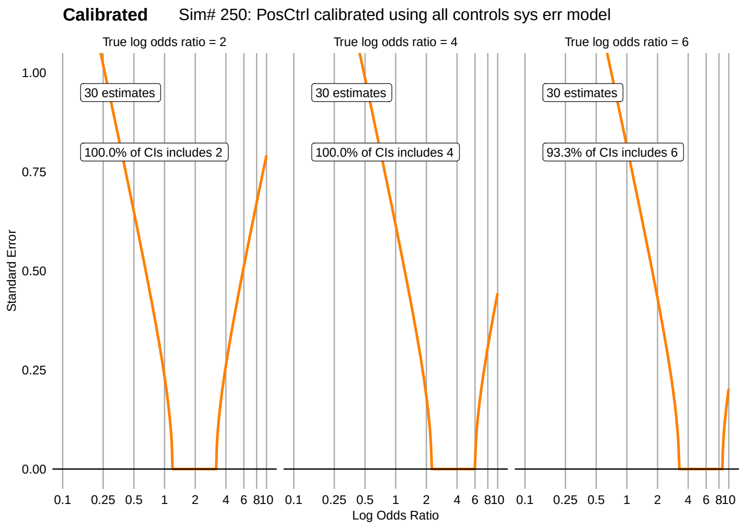

4.3 Interaction between Two Confounders

4.3.1 Calibration of Negative Controls (5)

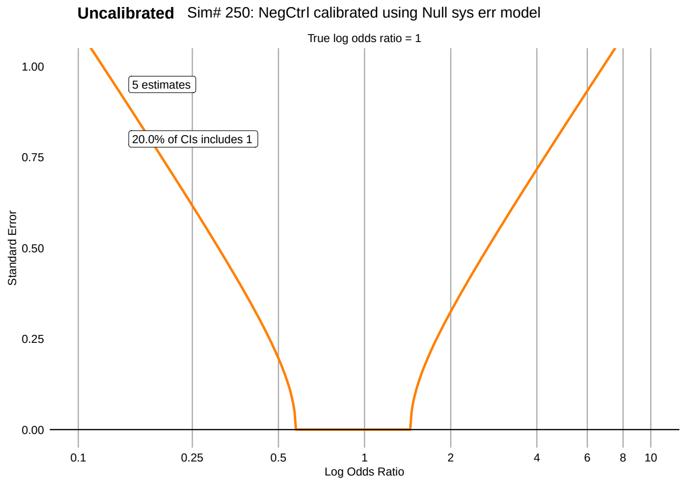

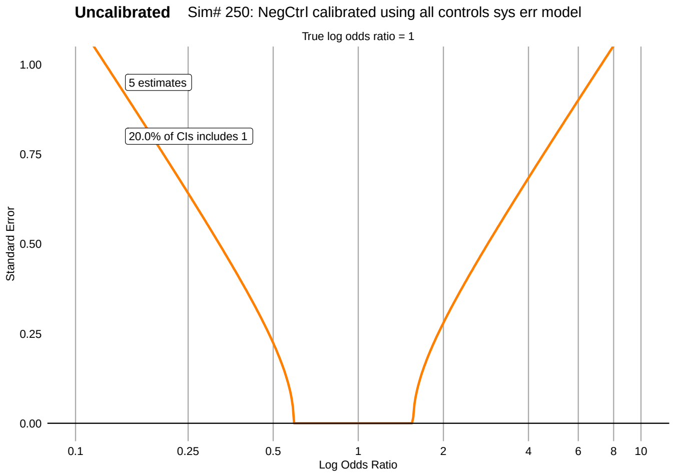

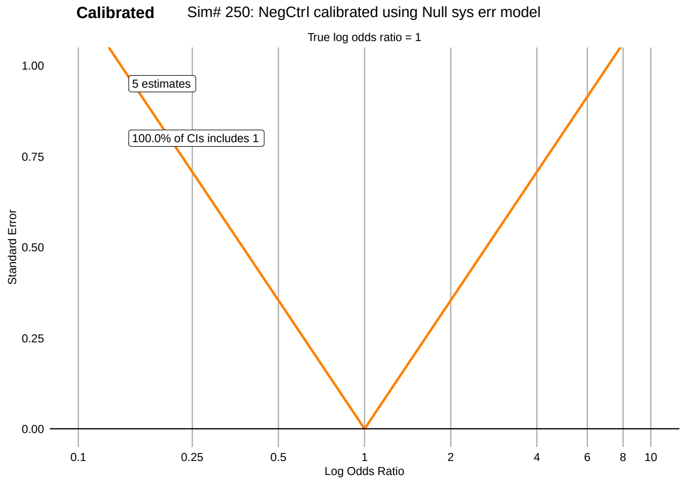

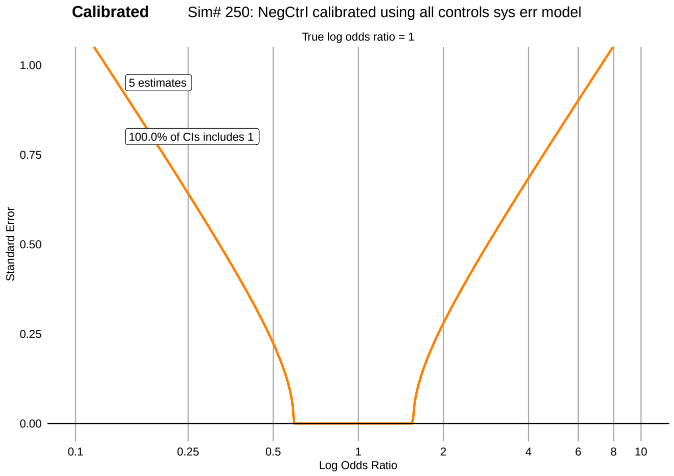

4.3.2 Calibration of Positive Controls (5)

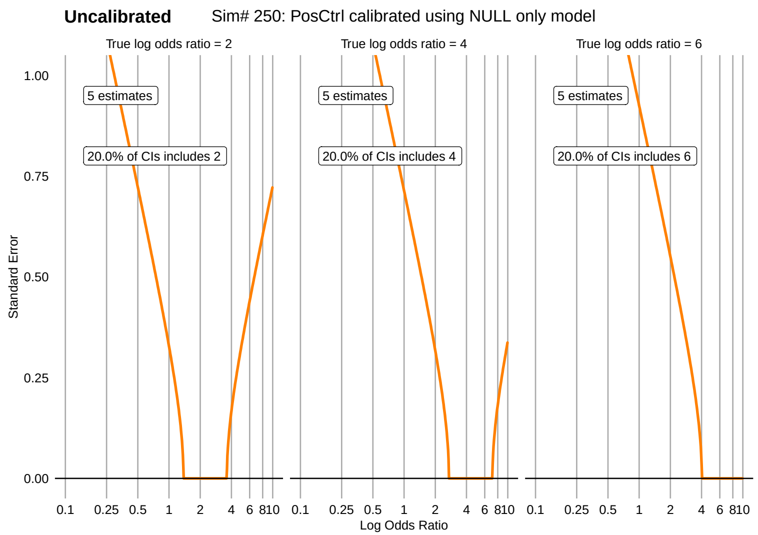

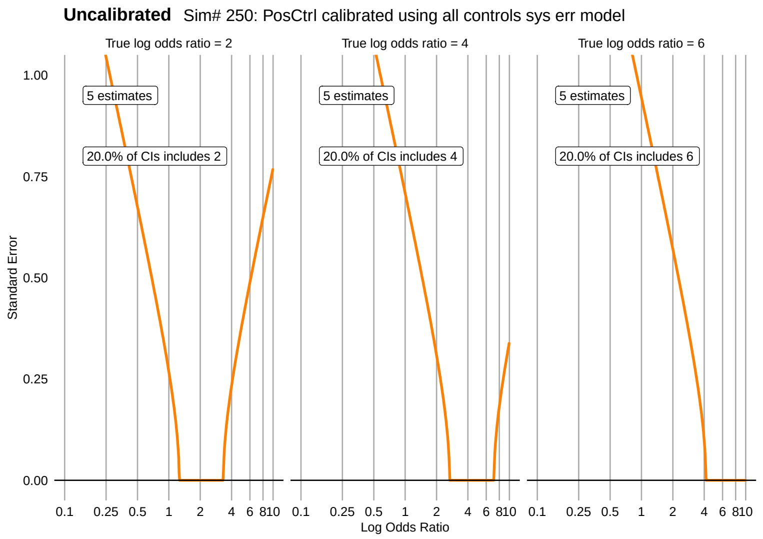

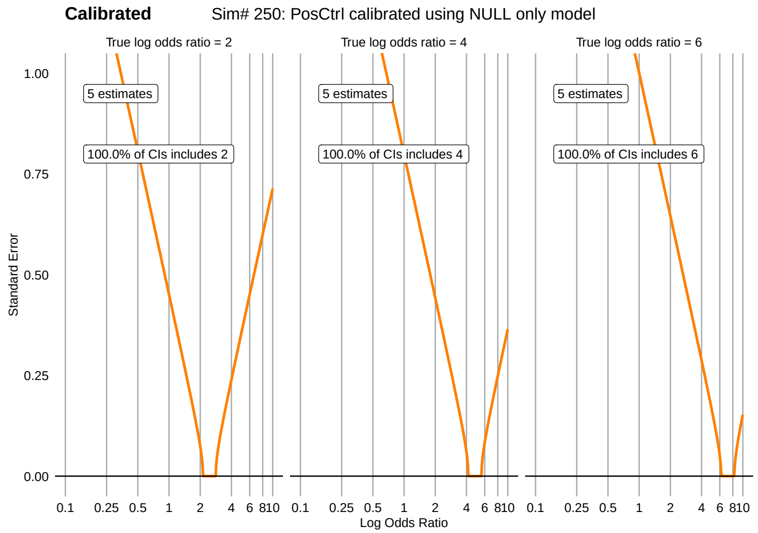

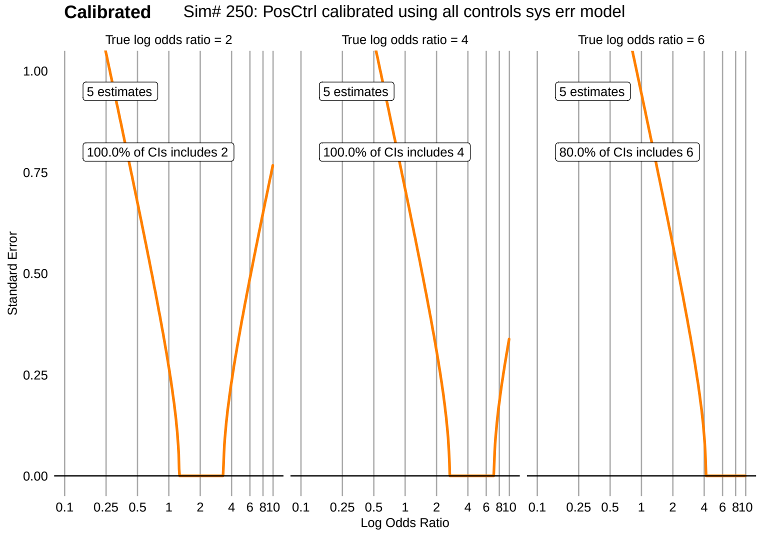

4.3.3 Calibration of Negative Controls (30)

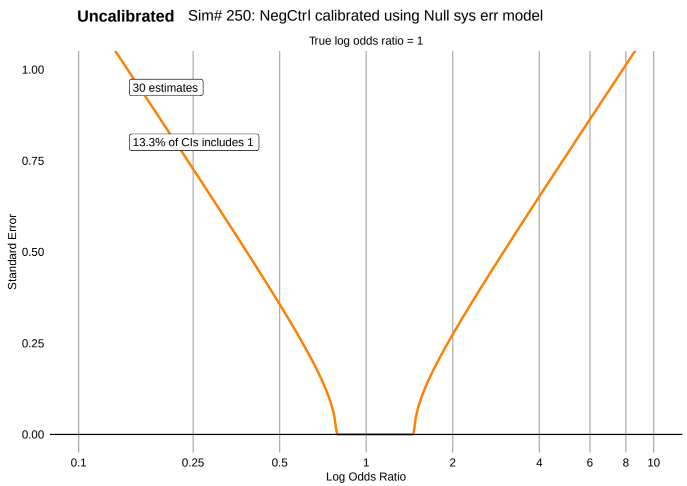

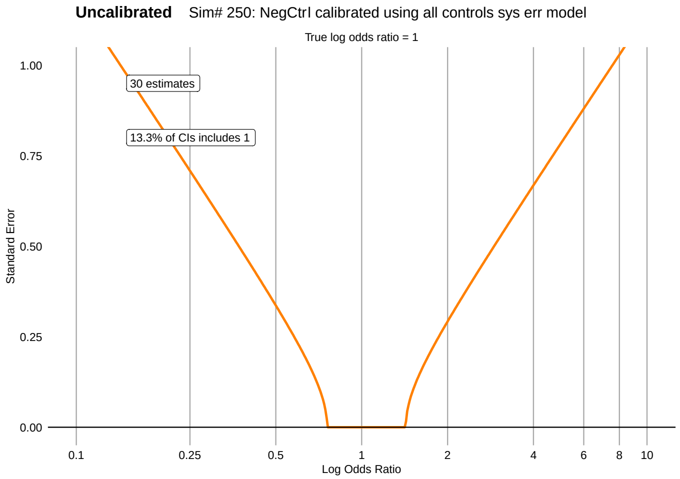

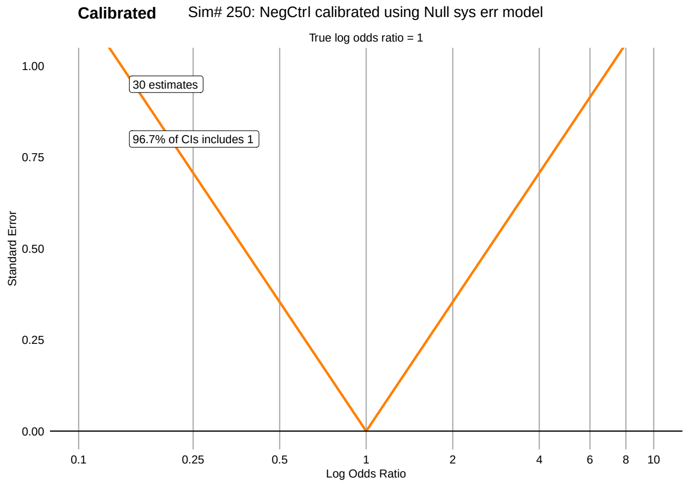

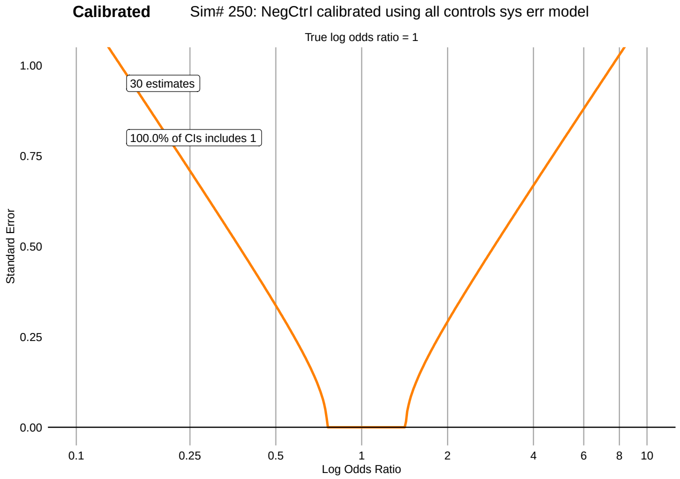

4.3.4 Calibration of Positive Controls (30)

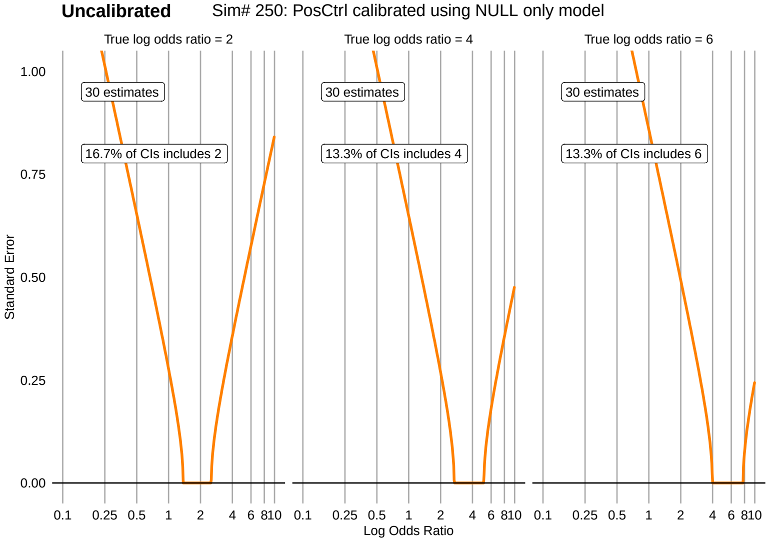

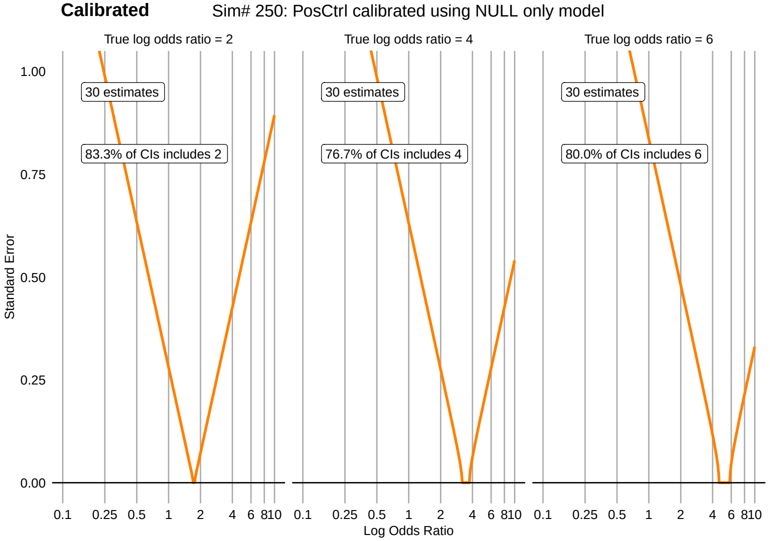

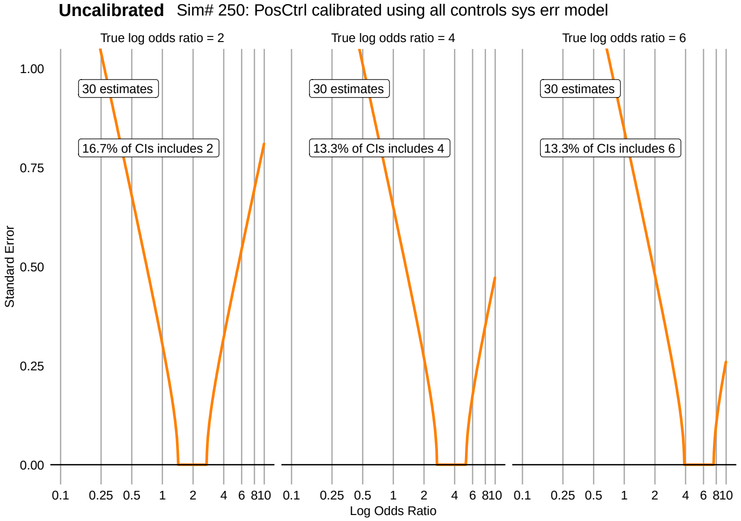

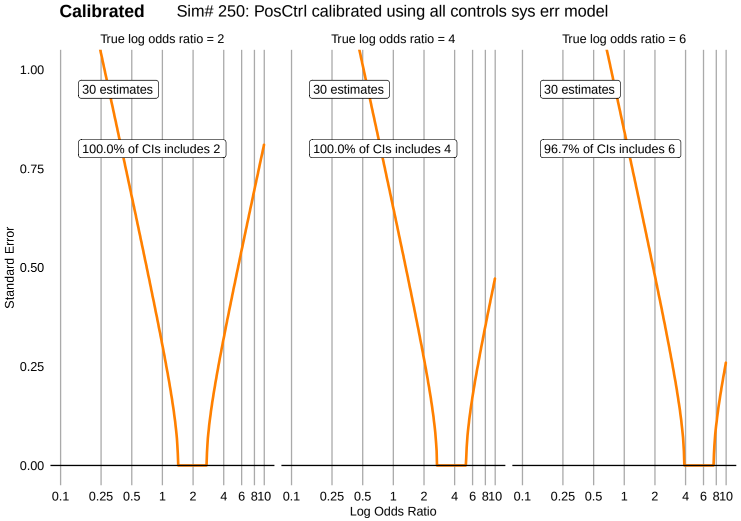

4.4 Lack of positivity scenario

4.4.1 Calibration of Negative Controls (5)

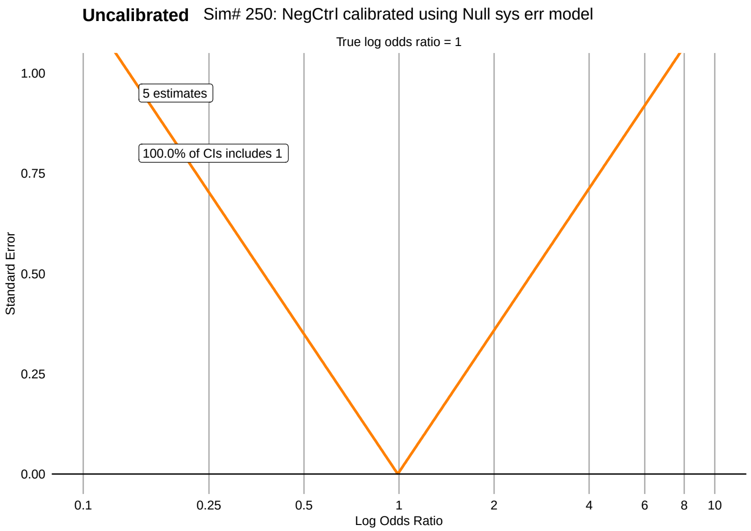

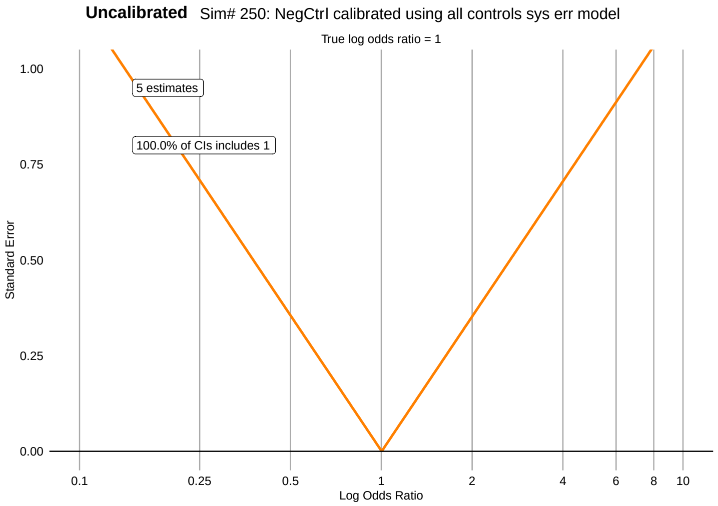

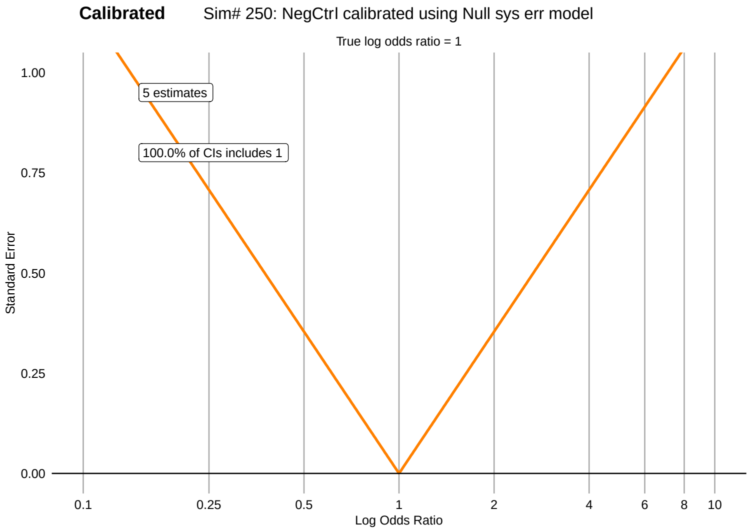

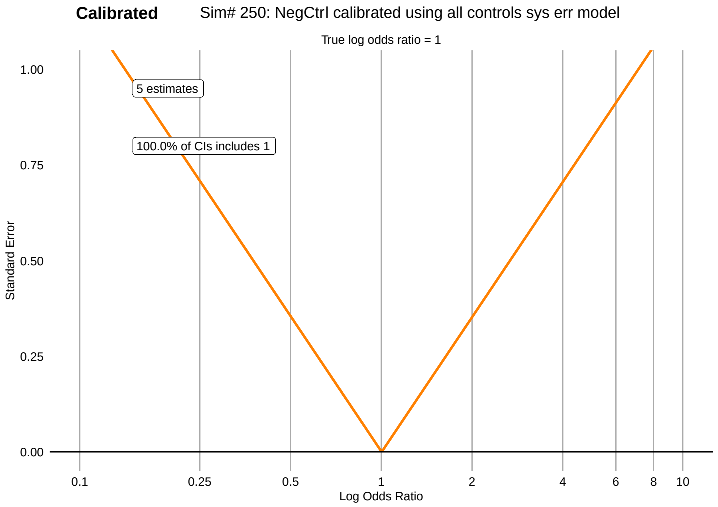

4.4.2 Calibration of Positive Controls (5)

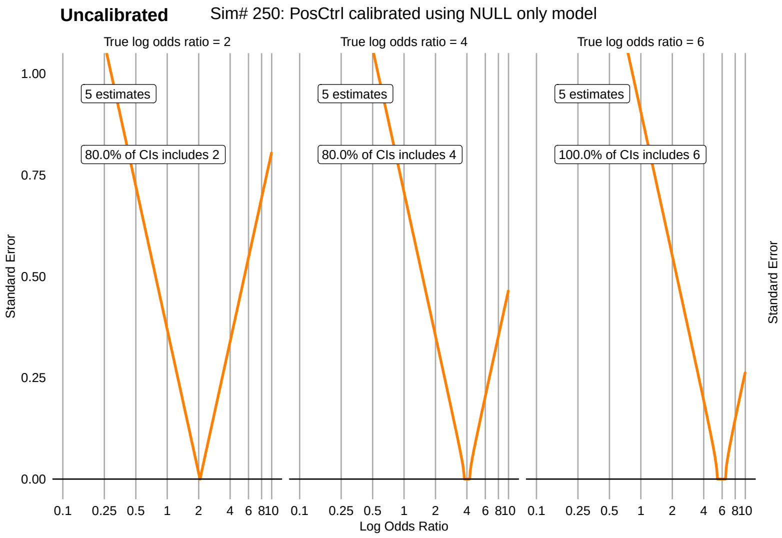

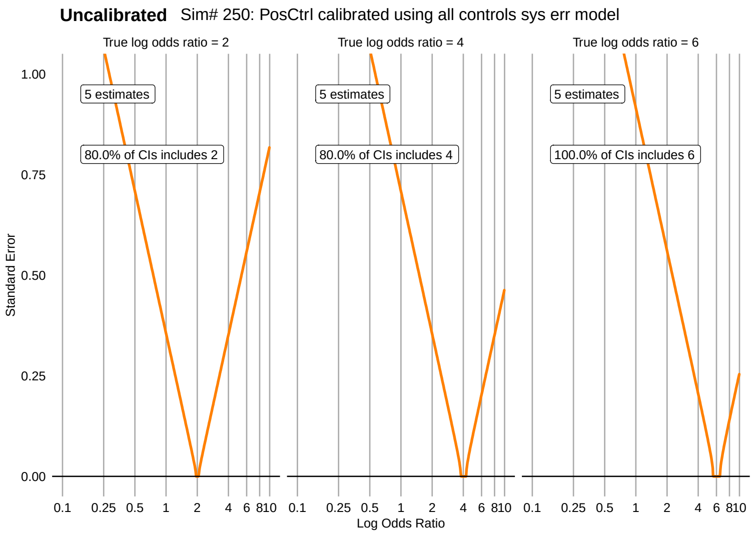

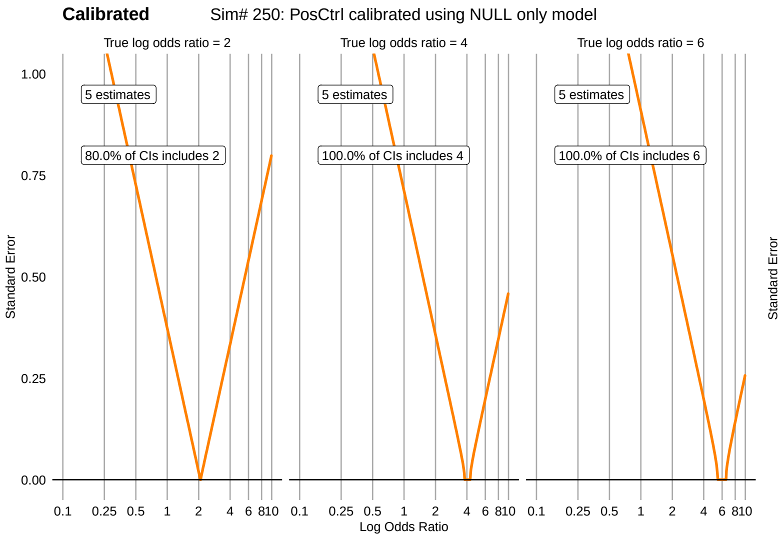

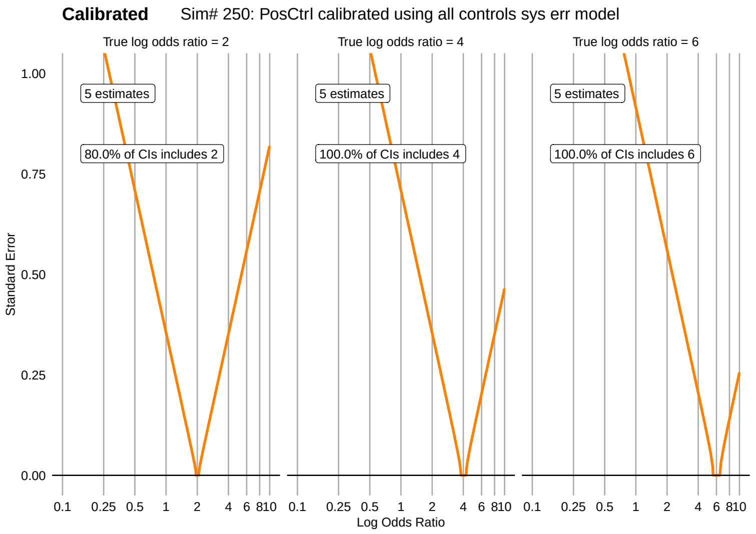

4.4.3 Calibration of Negative Controls (30)

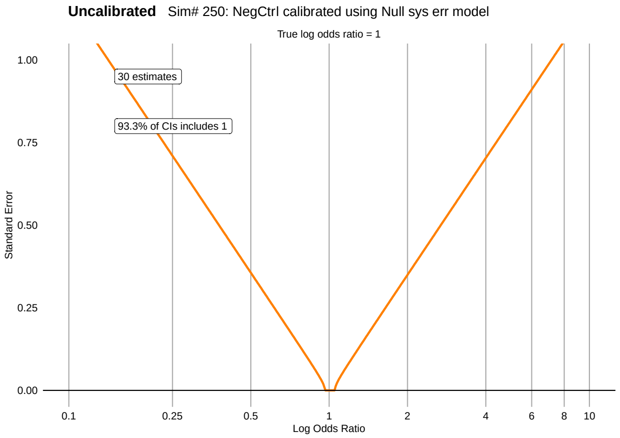

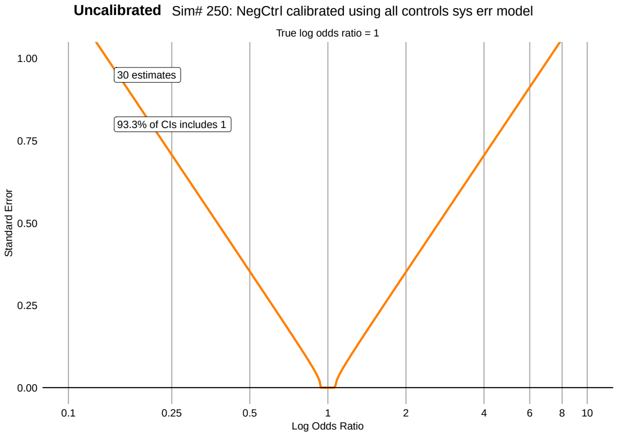

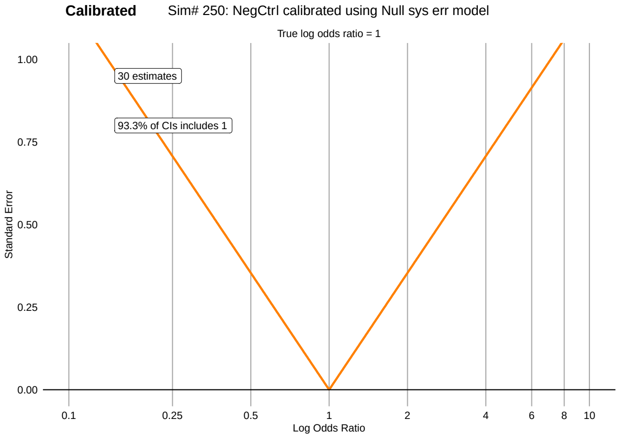

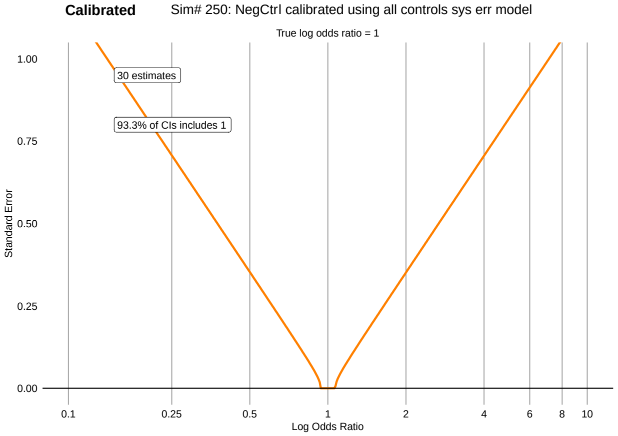

4.4.4 Calibration of Positive Controls (30)

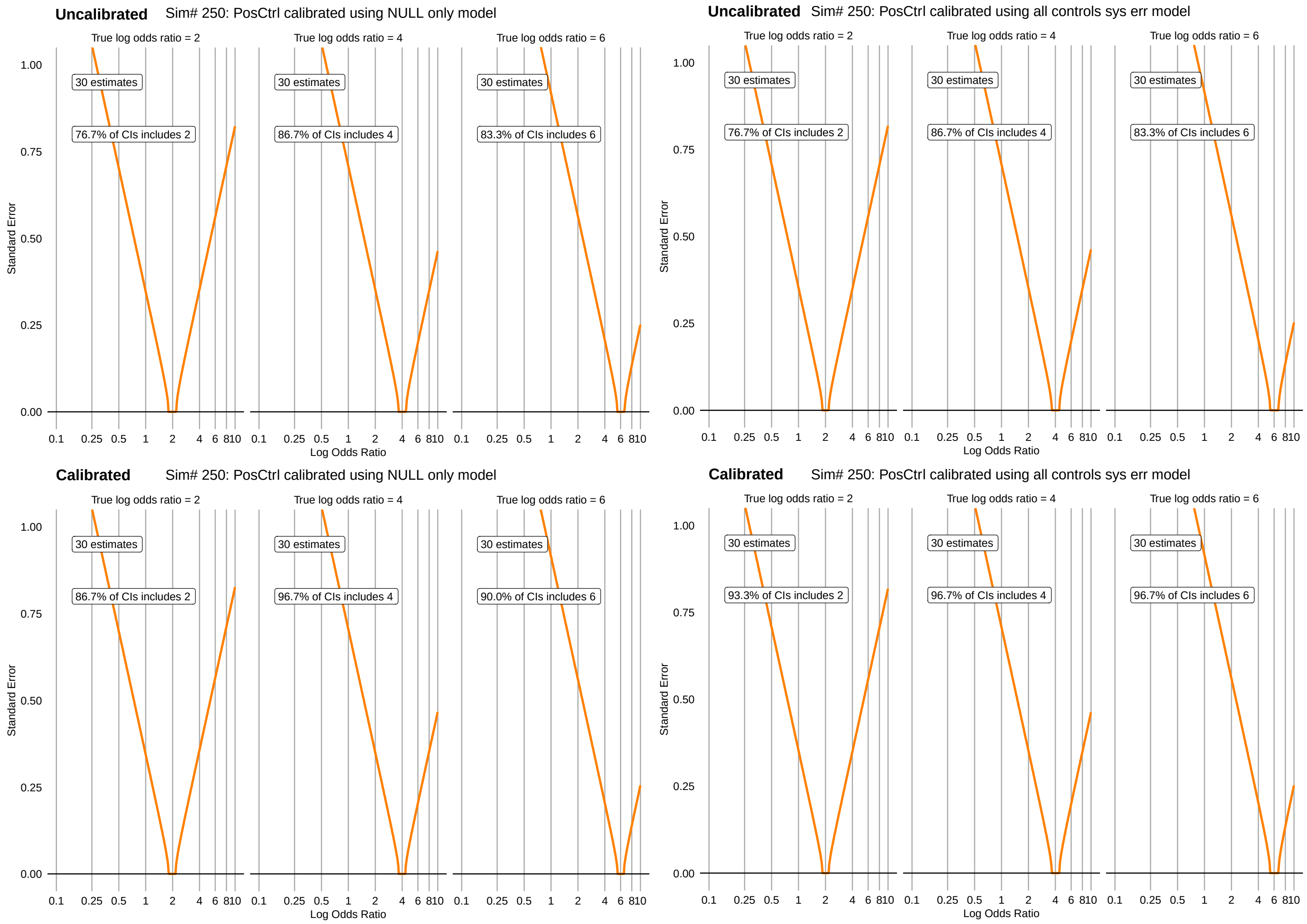

4.5 Measurement Error in confounder scenario

4.5.1 Calibration of Negative Controls (5)

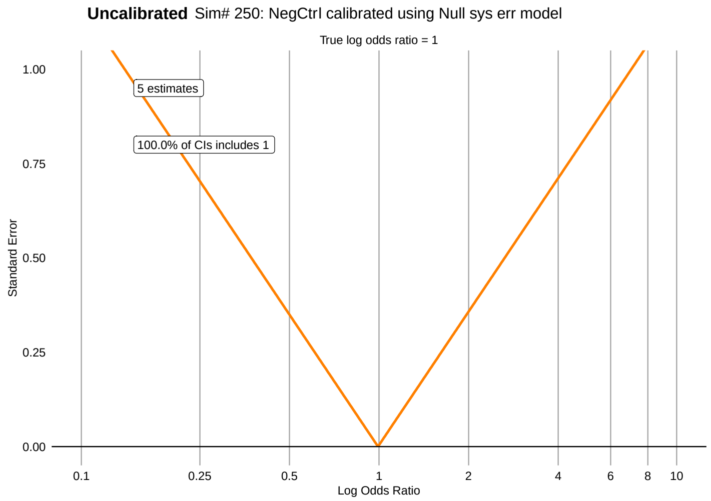

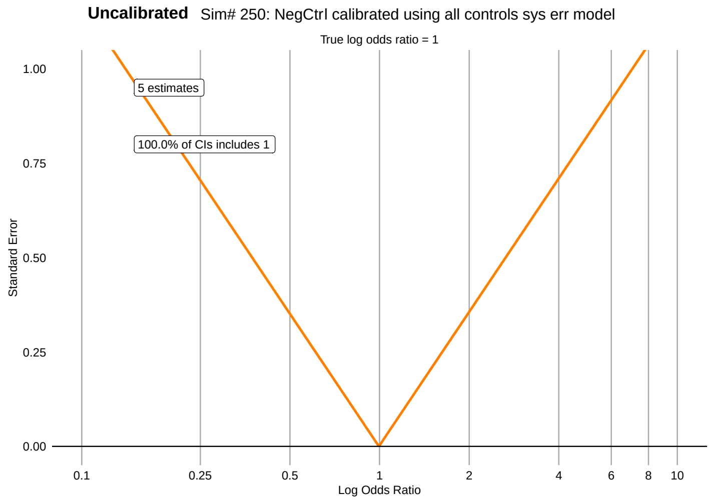

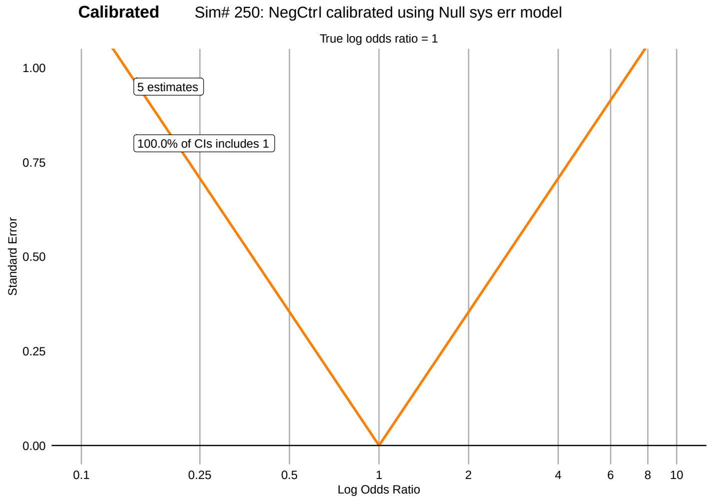

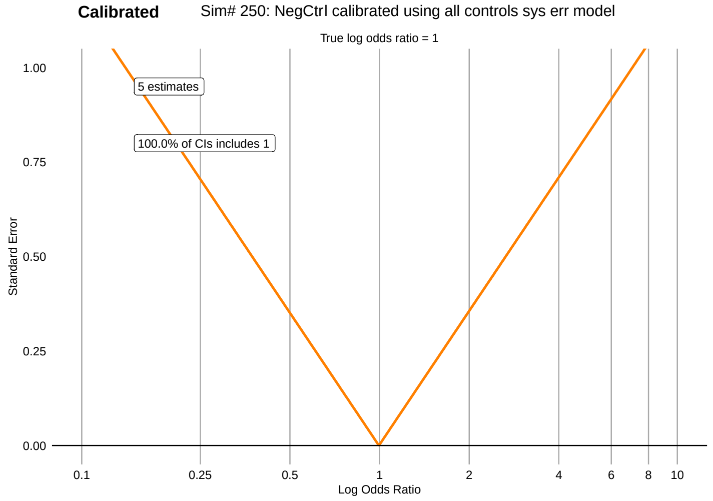

4.5.2 Calibration of Positive Controls (5)

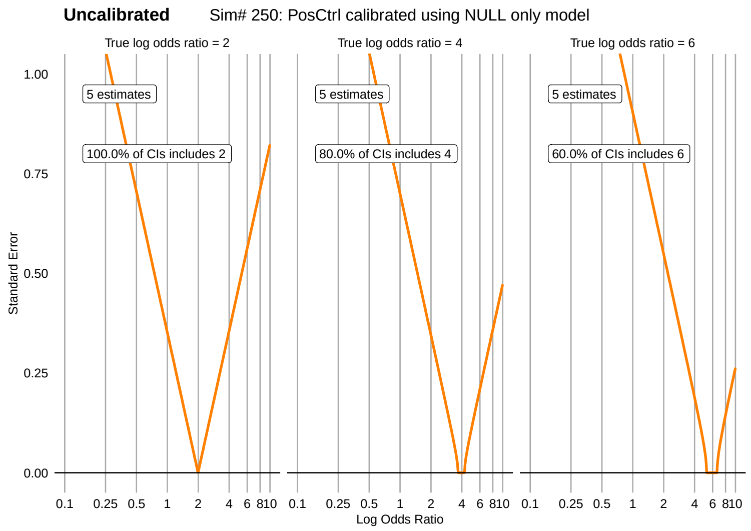

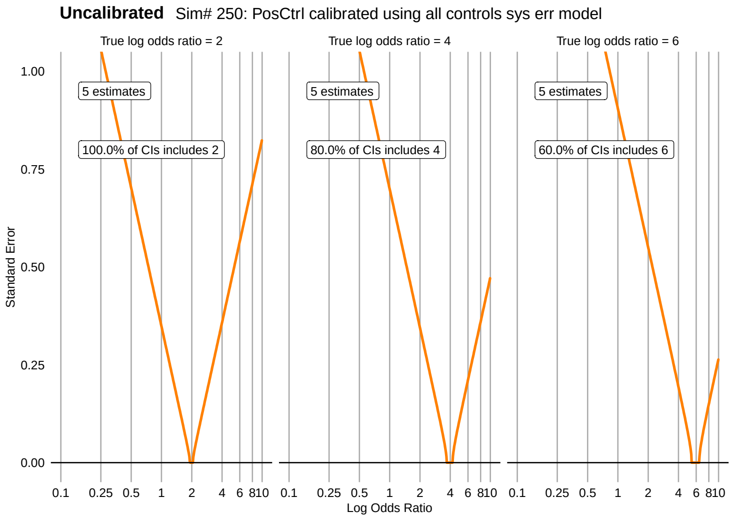

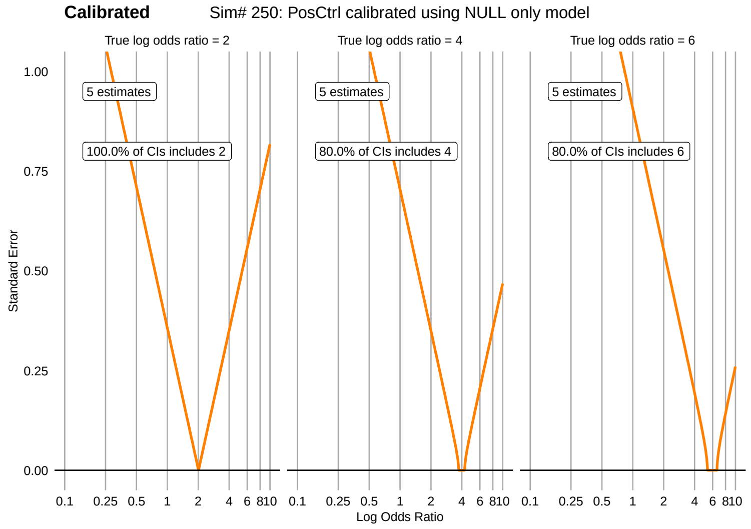

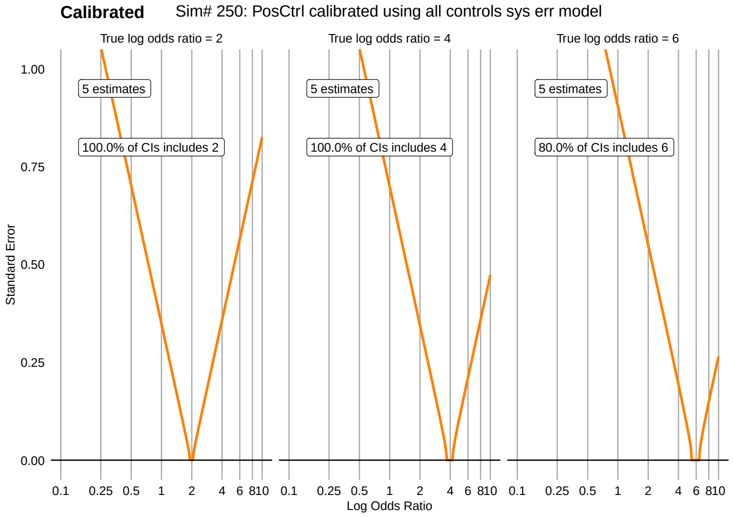

4.5.3 Calibration of Negative Controls (30)

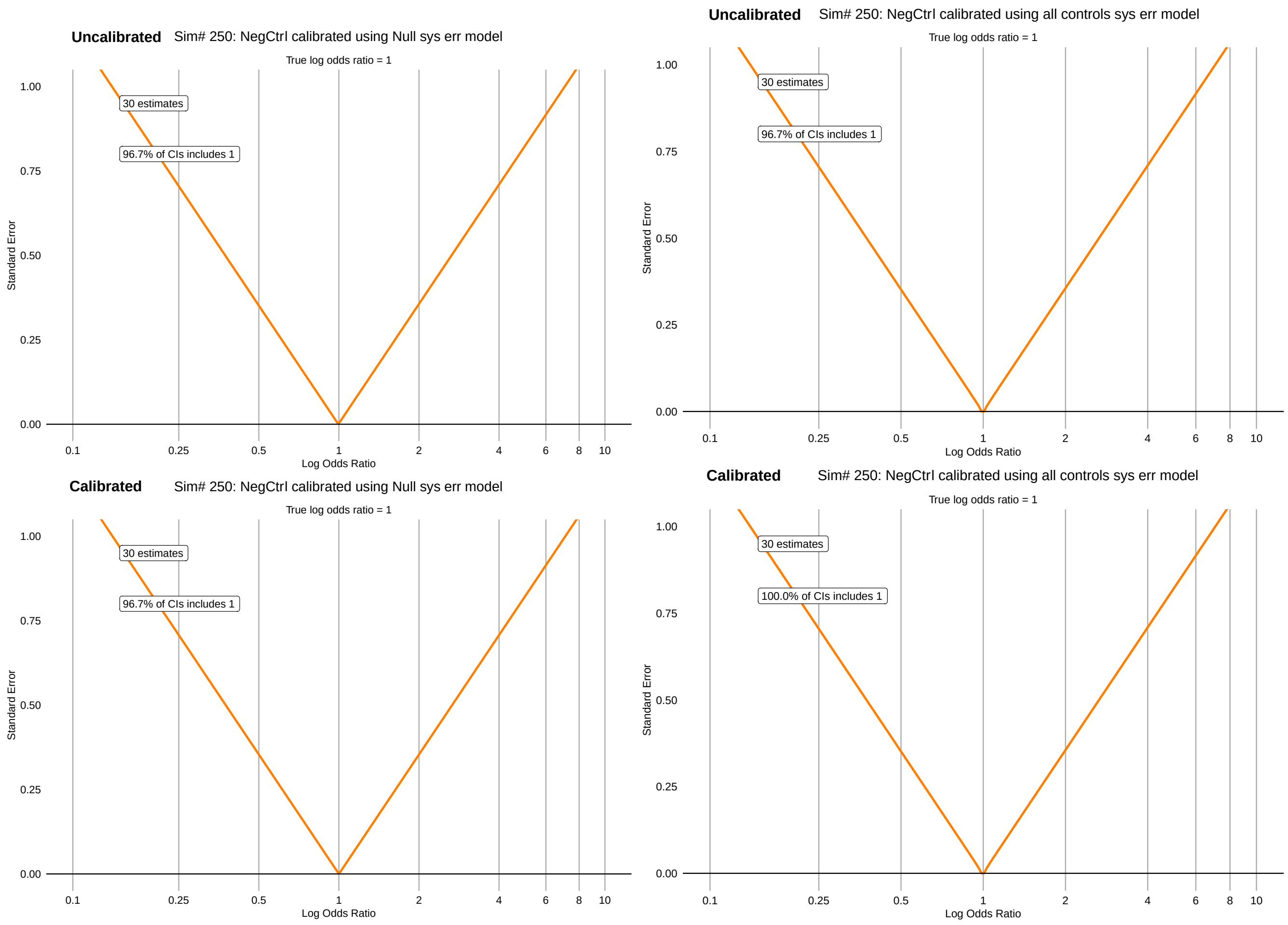

4.5.4 Calibration of Positive Controls (30)

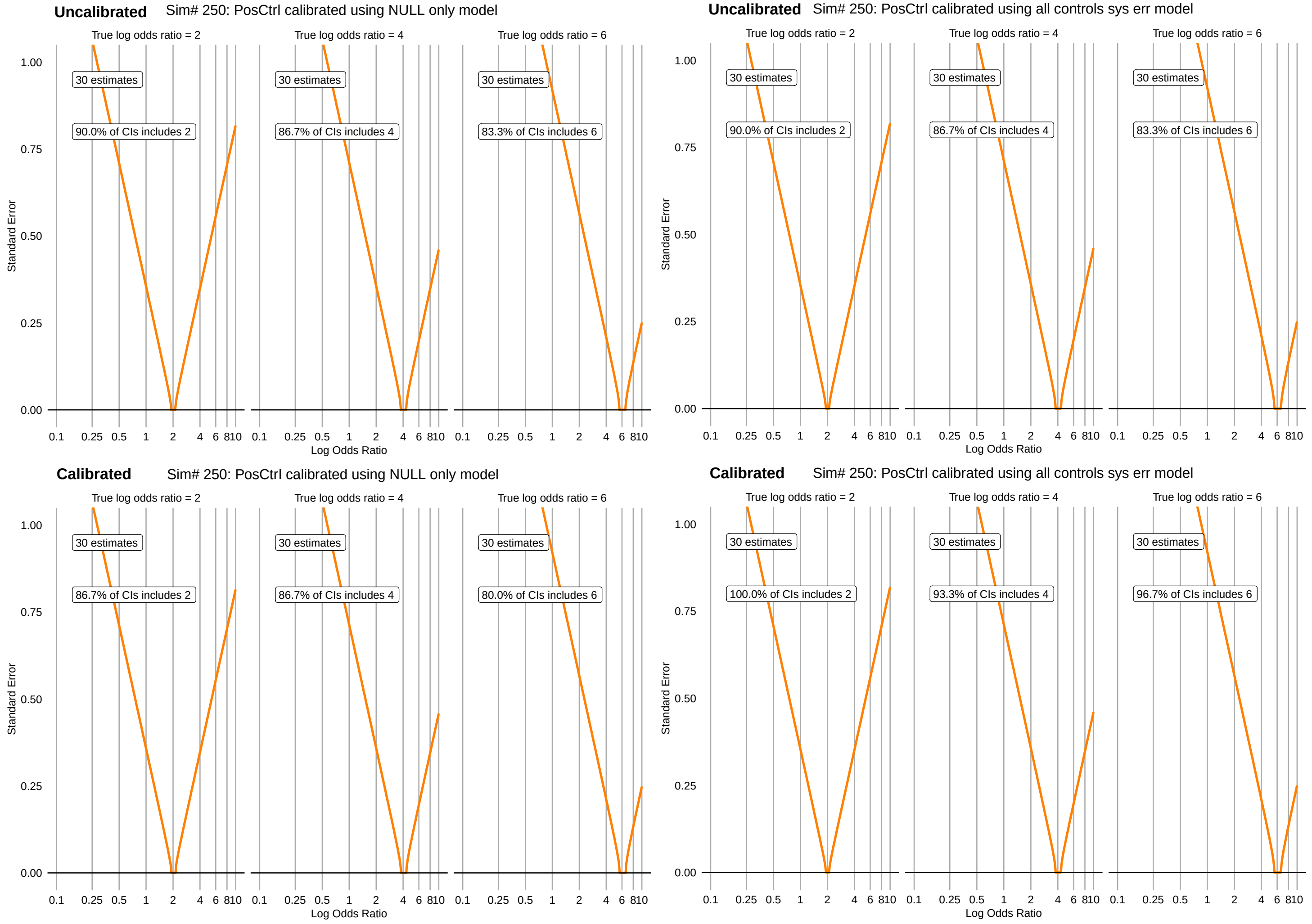

Supplement: Supplementary file 1 — Additional file 1. [file 12874_2022_1687_MOESM1_ESM.pdf]
